# Clinical outcomes of COVID-19 following the use of angiotensin-converting enzyme inhibitors or angiotensin-receptor blockers among patients with hypertension in Korea: a nationwide study

**DOI:** 10.4178/epih.e2021004

**Published:** 2020-12-29

**Authors:** Ju Hwan Kim, Yeon-Hee Baek, Hyesung Lee, Young June Choe, Hyun Joon Shin, Ju-Young Shin

**Affiliations:** 1 School of Pharmacy, Sungkyunkwan University, Suwon, Korea; 2 Division of Infectious Diseases, Department of Social and Preventive Medicine, Hallym University College of Medicine, Chuncheon, Korea; 3 Lemuel Shattuck Hospital, Boston, MA, USA; 4 Brigham and Women’s Hospital, Boston, MA, USA; 5 Department of Clinical Research Design and Evaluation, Samsung Advanced Institute for Health Sciences and Technology (SAIHST), Sungkyunkwan University, Seoul, Korea

**Keywords:** COVID-19, Hypertension, Angiotensin-converting enzyme inhibitors, Angiotensin-receptor blockers

## Abstract

**OBJECTIVES:**

Recent evidence has shown no harm associated with the use of angiotensin-converting enzyme inhibitors (ACEIs) or angiotensin-receptor blockers (ARBs) in patients with coronavirus disease 2019 (COVID-19). We sought to further clarify the possible association between ACEI/ARB use and the risk of poor clinical outcomes of COVID-19.

**METHODS:**

From the completely enumerated COVID-19 cohort in Korea, we identified 1,290 patients with hypertension, of whom 682 had and 603 did not have records of ACEI/ARB use during the 30-day period before their COVID-19 diagnosis. Our primary endpoint comprised clinical outcomes, including all-cause mortality, use of mechanical ventilation, intensive care unit admission, and sepsis. We used inverse probability of treatment weighting (IPTW) to mitigate selection bias, and a Poisson regression model to estimate the relative risks (RRs) and 95% confidence intervals (CIs) for comparing outcomes between ACEI/ARB users and non-users.

**RESULTS:**

Compared to non-use, ACEI/ARB use was associated with lower clinical outcomes (IPTW-adjusted RR, 0.60; 95% CI, 0.42 to 0.85; p=0.005). For individual outcomes, ACEI/ARB use was not associated with all-cause mortality (IPTW-adjusted RR, 0.62; 95% CI, 0.35 to 1.09; p=0.097) or respiratory events (IPTW-adjusted RR, 0.99; 95% CI, 0.84 to 1.17; p=0.904). Subgroup analysis showed a trend toward a protective role of ACEIs and ARBs against overall outcomes in men (IPTW-adjusted RR, 0.84; 95% CI, 0.69 to 1.03; p_interaction_=0.008) and patients with pre-existing respiratory disease (IPTW-adjusted RR, 0.74; 95% CI, 0.60 to 0.92; p_interaction_=0.002).

**CONCLUSIONS:**

We present clinical evidence to support continuing ACE/ARB use in COVID-19 patients with hypertension based on the completely enumerated Korean cohort.

## INTRODUCTION

Coronavirus disease 2019 (COVID-19), caused by severe acute respiratory syndrome coronavirus 2 (SARS-CoV-2), has rapidly spread throughout the world and remains an ongoing pandemic [1]. Concerns have been raised regarding the possibility that a poor prognosis of COVID-19 may be associated with the use of angiotensin-converting enzyme inhibitors (ACEIs) and angiotensin-receptor blockers (ARBs) [2]. ACEIs and ARBs have been shown to upregulate angiotensin-converting enzyme 2 (ACE2) expression and activity in several experimental studies [3-5]. Given that binding of ACE2 with the viral spike protein of SARS-CoV-2 allows the virus to enter host cells, it was hypothesized that the potential upregulation of ACE2 may lead to an increased severity of illness or risk of mortality in COVID-19 patients [2].

In theory, patients with hypertension managed with ACEIs or ARBs could be at an increased risk of a poor prognosis from COVID-19 since the increased expression of ACE2 caused by these drug classes may increase viral entry into cells. Alternatively, increased ACE2 expression has been recognized to counterbalance the pro-inflammatory and vasoconstrictive effect of ACE, mainly through conversion of angiotensin II (Ang II) to Ang-(1-7), a peptide with potential protective anti-inflammatory properties that counterbalances the pro-inflammatory activity of Ang II [6-8].

In view of these 2 opposing mechanistic hypotheses, the demand for clinical research on this topic remains very high. To date, several observational studies have claimed that the use of ACEIs/ARBs was not associated with increased all-cause mortality [9-13]. However, these studies were limited due to methodological issues in their study design, with some lacking an assessment of a causal relationship [9-12], and the other suffering from immortal time bias arising from misclassification of the exposure period [13]. Given the lack of a robust population-based study assessing the association between the use of ACEIs/ARBs and the outcomes of COVID-19, we analyzed patients with hypertension from the completely enumerated COVID-19 cohort in Korea to assess whether the use of ACEIs and ARBs was associated with poor clinical outcomes of COVID-19.

## MATERIALS AND METHODS

We retrieved the healthcare database from the Health Insurance Review and Assessment Service of Korea, which covers the entire Korean population (over 50 million people), from January 1, 2015 to April 8, 2020. We used the completely enumerated database of 69,793 subjects who underwent COVID-19 screening tests in Korea. The database contains both inpatient and outpatient prescriptions, demographic characteristics (age, sex, and insurance type), and clinical information on visit dates for hospitalization and ambulatory care, procedures, and diagnosis records coded using the Korean Standard Classification of Diseases, seventh revision, which is based on the International Classification of Diseases, 10th revision. The overall agreement of the diagnostic records of hypertension, stroke, and heart disease was 93.73%, 98.80%, and 97.93%, respectively [14].

We conducted a retrospective cohort study of ACEI/ARB use and adverse outcomes of COVID-19 among patients with hypertension. We identified patients with laboratory-confirmed diagnoses of COVID-19 between December 1, 2019 and April 8, 2020. Diagnoses were made based on the diagnostic results from reversetranscription polymerase chain reaction targeting the RNA-dependent RNA polymerase, N, and E genes as recommended by the interim guidance of World Health Organization [15]. Cohort entry was defined as the date of incident diagnosis of COVID-19. We required patients to have a recorded diagnosis of hypertension within a 5-year lookback period from cohort entry.

Exposure to ACEIs and ARBs was ascertained within 30 days preceding cohort entry. Our exposure of interest was patients who had ever been prescribed ACEIs or ARBs, either as monotherapy or combination therapy. Non-users were those who had no prescription record of either ACEIs or ARBs during the exposure ascertainment period.

We investigated clinical outcomes indicative of a poor COVID-19 prognosis, and the primary endpoint was a composite outcome comprising all-cause mortality, use of mechanical ventilation, intensive care unit (ICU) admission, or sepsis. We also assessed all-cause mortality and respiratory events (acute respiratory distress syndrome, interstitial lung disease, pneumonia, and respiratory failure) individually as secondary endpoints. Each patient was followed until the occurrence of the outcome of interest or the data-censoring date.

We assessed baseline characteristics within 1 year before cohort entry. To generate a propensity score, we used baseline confounders including age at cohort entry, sex, income level, the CHA₂DS₂- VASc score (a validated risk stratification tool for predicting stroke in patients with atrial fibrillation, as well as morbidity and mortality in several disease categories), medical history (including diabetes, cardiovascular disease [CVD], stroke, other cerebrovascular disease, hyperlipidemia, respiratory disease, chronic kidney disease, cancer, thromboembolism, and dementia), comedications including other antihypertensives (calcium channel blockers, diuretics, β-blockers, and α-blockers), antidiabetics, antibiotics, antiarrhythmics, antiplatelets, anticoagulants, lipid-lowering agents, and antianginal agents, dialysis, and the duration of hypertension (< 1, ≥ 1 and < 3, ≥ 3 and < 5, and ≥ 5 years).

We estimated propensity scores for receiving ACEIs or ARBs by fitting a multivariable logistic regression model using all predefined covariates assessed 1 year before cohort entry. We used inverse probability of treatment weighting (IPTW) based on the propensity scores to mitigate selection bias based on different characteristics between ACEI/ARB users and non-users. IPTW creates a pseudo-population, where the weighted exposure and comparator groups are representative of the patient characteristics in the overall population, and thus generates the population-average treatment effect [16]. We summarized the baseline characteristics of the study cohort using counts and proportions or mean values for categorical or continuous variables, respectively. We used a Poisson regression model to estimate relative risk (RR) and the corresponding 95% confidence intervals (CIs) for each outcome in ACEI/ARB users compared to non-users among patients with COVID-19. The unweighted model was adjusted for predefined covariates including age, sex, CHA₂DS₂-VASc score, diabetes, CVD, and baseline respiratory diseases for parsimony. These covariates were also used in the IPTW models for a doubly robust estimation of the causal effect. We chose the adjusted IPTW model as the main model to report the RRs and corresponding 95% CIs for clinical outcomes in ACEI/ARB users, compared with non-users.

Given that ACEIs and ARBs, apart from hypertension, are primarily prescribed for patients with diabetes and CVD, we repeated the analysis with a restricted cohort of patients with these health conditions to exclude confounding by indication. To evaluate whether the association differed by patients’ underlying conditions, we conducted additional subgroup analyses using the interaction terms by age group, sex, CHA₂DS₂-VASc score, pre-existing respiratory disease, and hospitalization after diagnosis of COVID-19. In the subgroup analyses, we used overall outcomes, which included all-cause mortality, use of mechanical ventilation, ICU admission, sepsis, or the occurrence of respiratory events to increase the statistical power. Additionally, we conducted a sensitivity analysis where we redefined the exposure assessment window to be within 180 days preceding cohort entry to address potential exposure misclassification. All statistical analyses were performed using the SAS Enterprise Guide version 6.1 (SAS Institute Inc., Cary, NC, USA), and a 2-sided α of less than 0.05 was considered to indicate statistical significance.

### Ethics statement

The study protocol was approved by the Institutional Review Board (IRB) of Sungkyunkwan University (SKKU 2020-03-021) and the requirement to obtain informed consent was waived by the IRB.

## RESULTS

**Figure 1.**
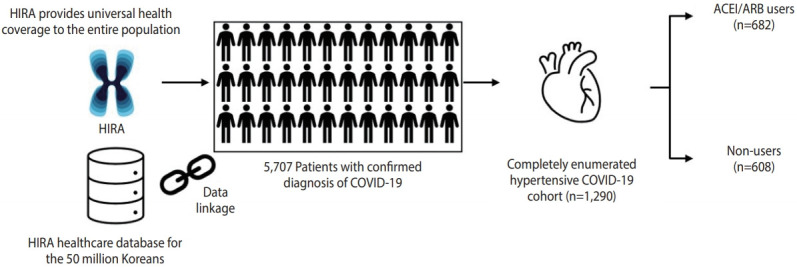
Description of data source and selection of the cohort of coronavirus disease 2019 (COVID-19) patients with hypertension. HIRA, Health Insurance Review and Assessment Service; ACEI, angiotensin-converting enzyme inhibitor; ARB, angiotensin II receptor blocker.

Among 5,707 patients with a confirmed diagnosis of COVID-19, there were 1,290 patients with a past medical history of hypertension, of whom 682 had records of ACEI/ARB use and 608 did not have records of ACEI/ARB use during 30 days preceding cohort entry (Figure 1). The characteristics of the ACEI/ARB users compared with non-users are described in Table 1. Compared to nonusers, ACEI/ARB users were older (mean± standard deviation, 62.8± 14.4 vs. 61.3± 16.6 years), had a higher proportion of male (53.4 vs. 49.8%), and had a higher prevalence of hyperlipidemia (38.6 vs. 33.6%), diabetes (37.0 vs. 25.7%), CVD (27.9 vs. 26.0%), chronic kidney disease (18.8 vs. 15.6%), and duration of hypertension over 5 years (56.7 vs. 41.5%). The concomitant use of other anti-hypertensives was generally similar between ACEI/ARB users and non-users, while the mean CHA_2_DS_2_-VASc score was higher in ACEI/ARB users (2.7 vs. 2.4).

During the study period, there were 23 (3.4%) and 28 (4.6%) cases of clinical outcomes in ACEI/ARB users and non-users, respectively (Table 2). Compared to non-use, ACEI/ARB use was associated with a lower likelihood of clinical outcomes, including all-cause mortality, mechanical ventilation, ICU admission, and sepsis (IPTW-adjusted RR, 0.60; 95% CI, 0.42 to 0.85; p= 0.005). Regarding individual outcome events, ACEI/ARB use was not associated with the risk of all-cause mortality (IPTW-adjusted RR, 0.62; 95% CI, 0.35 to 1.09; p= 0.097) and respiratory events (IPTW-adjusted RR, 0.99; 95% CI, 0.84 to 1.17; p= 0.904) compared with non-use.

We conducted a sensitivity analysis where we redefined the exposure risk window as 180 days preceding cohort entry to account for potential exposure misclassification (Table 3). There were 28 (3.7%) and 23 (4.4%) cases of adverse outcomes in ACEI/ARB users and non-users, respectively. The results from the sensitivity analysis were generally consistent with the main analysis; ACEI/ARB use was associated with a lower likelihood of clinical outcomes (IPTW-adjusted RR, 0.65; 95% CI, 0.46 to 0.90; p= 0.009) and all-cause mortality (IPTW-adjusted RR, 0.41; 95% CI, 0.25 to 0.68; p = 0.001), but was not associated with respiratory events (IPTW-adjusted RR, 0.94; 95% CI, 0.81 to 1.09; p= 0.419) compared with non-use.

**Figure 2.**
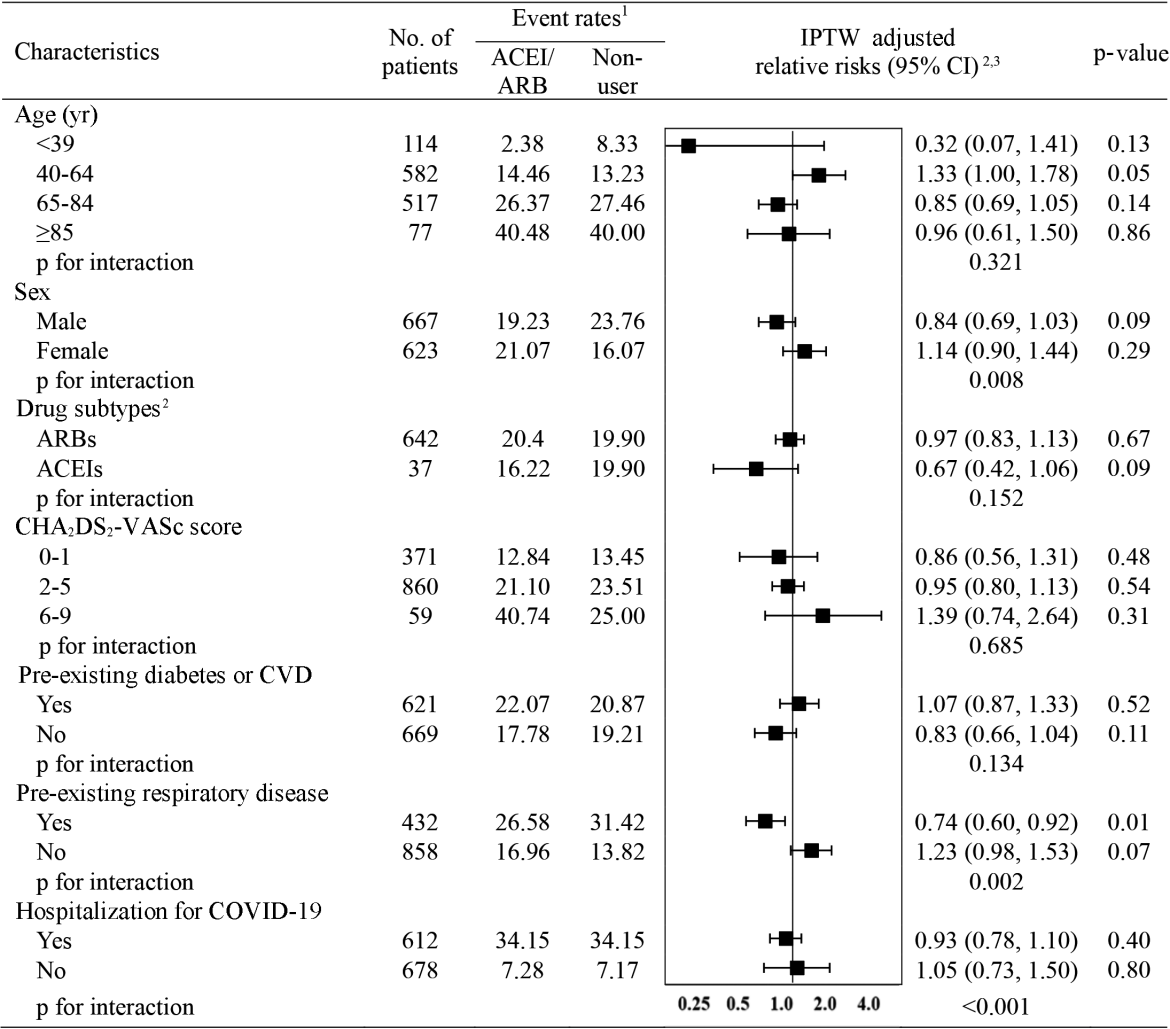
Relative risks (95% CIs) of overall adverse outcome events in ACEI/ARB users compared to non-users in selected population subgroups. ACEIs, angiotensin-converting enzyme inhibitors; ARBs, angiotensin receptor blockers; CI, confidence interval; IPTW, inverse probability of treatment weighting; CVD, cardiovascular disease; COVID-19, coronavirus disease 2019. ^1^Overall adverse outcome events include all-cause mortality, use of mechanical ventilation, admission to intensive care unit, sepsis, or the occurrence of respiratory events. ^2^Relative risks were adjusted for age, sex, CHA₂DS₂-VASc score, diabetes, CVD, and respiratory disease. ^3^To make IPTW, age at cohort entry, sex, income level, CHA₂DS₂-VASc score, medical history (including diabetes, CVD, stroke, other cerebrovascular disease, hyperlipidemia, respiratory disease, chronic kidney disease, cancer, thromboembolism, and dementia), comedications (including other antihypertensives [calcium channel blockers, diuretics, β-blockers, and alpha blockers], antidiabetics, antibiotics, antiarrhythmics, antiplatelets, anticoagulants, lipidlowering agents, and antianginal agents), dialysis, and the duration of hypertension (<1, ≥1 and <3, ≥3 and <5, and ≥5 years).

The subgroup analysis of the risk of clinical outcomes compared with non-use is presented in Figure 2. When assessed by exposure subtype, no significant interaction was found between the subtypes and the overall outcomes (p_interaction_= 0.015); neither ACEI (IPTW-adjusted RR, 0.67; 95% CI, 0.42 to 1.06) nor ARB use (IPTW-adjusted RR, 0.97; 95% CI, 0.83 to 1.13) was associated with the risk of overall adverse outcomes. Interestingly, interaction-term analysis showed a trend toward a protective role of ACEIs and ARBs against overall outcomes in male (IPTW-adjusted RR, 0.84; 95% CI, 0.69 to 1.03; p_interaction_= 0.008), patients with pre-existing respiratory disease (IPTW-adjusted RR, 0.74; 95% CI, 0.60 to 0.92; p_interaction_= 0.002), and patients hospitalized for COVID-19 (IPTW-adjusted RR, 0.93; 95% CI, 0.78 to 1.10; p_interaction_ < 0.001).

## DISCUSSION

We used medical claims data of patients diagnosed with COVID-19 in Korea to demonstrate that ACEI/ARB use was not associated with poor clinical outcomes from COVID-19 among patients with hypertension. Specifically, ACEI/ARB use, compared with non-use, was associated with a lower likelihood of the composite clinical outcome that comprised all-cause mortality, use of mechanical ventilation, ICU admission, and sepsis. To account for exposure misclassification, we conducted a sensitivity analysis to assess exposure status during a period of 180 days preceding cohort entry, and the IPTW-adjusted RRs were largely consistent with the findings from main analysis across all outcome measures. Furthermore, the results of the subgroup analysis accounting for potential confounding by indication also remained largely consistent with the findings from main analysis.

While the underlying pathogenic link between hypertension and COVID-19 remains to be elucidated, concerns have been raised that ACEIs and ARBs, mainstays of therapy for hypertension and diabetes, may contribute to the adverse outcomes observed in COVID-19 patients [2]. Indeed, interaction between SARS-CoV-2 and ACE2 was proposed as a potential mechanism for the entry of SARS-CoV-2 into the cell [17], and the administration of ACEIs and ARBs upregulated ACE2 expression and activity in several experimental studies [3-5], implying that patients on ACEIs or ARBs may theoretically be exposed to a greater risk from COVID-19. Conversely, a beneficial role of ACE2 in COVID-19 has been reported, as a recent pilot clinical trial in patients with acute respiratory distress syndrome demonstrated the promising role of recombinant human ACE2 in attenuating the acute lung injury [18]. Moreover, experimental evidence was found that ARBs, specifically losartan, restored the expression level of ACE2, which was downregulated in preclinical models of experimental SARS-CoV infection and acute lung injury [3,19,20]. While debate continues on whether to continue or halt ACEIs/ARBs in COVID-19 patients with hypertension, the real-world data from our study complement the position statements made by medical societies such as European Society of Cardiology Council, American College of Cardiology, American Heart Association, and Heart Failure Society of America on continuing the use of ACEIs or ARBs as prescribed [21,22], as ACEI/ARB use was not associated with poor clinical outcomes of COVID-19.

Consistent with our findings, several recently published studies also have demonstrated no harm or even a protective role of ACEIs/ARBs in COVID-19. A study in Italy utilized a case-control design with 6,272 COVID-19 cases and 30,579 matched controls and reported that ACEI/ARB use was not associated with the risk of COVID-19 (adjusted odds ratio, 0.95; 95% CI, 0.86 to 1.05) [11]. The most recent single tertiary center-based study in the United States also reported no association between ACEI/ARB use and poor outcomes of COVID-19 among 2,573 COVID-19 patients with hypertension; the median difference in percentage points between ACEI/ARB users and non-users was -0.5% (95% CI, -4.3 to 3.2) [12]. Although the methodological issues inherent in observational studies limit the interpretation of the study findings, the conclusions of these recently published studies are consistent with the findings of our study, and provide clinical evidence that ACEI/ARB use is not associated with an increased risk of poor clinical outcomes from COVID-19. Supplementing earlier observations of a protective role of ACEIs and ARBs in COVID-19, our subgroup analysis showed a greater benefit with regard to clinical outcomes from COVID-19 in association with ACEI/ARB use than with non-use in males, in patients with pre-existing respiratory disease, and in patients hospitalized for COVID-19. These subgroups have been reported to have poor prognoses of COVID-19 [11], and our study findings should be interpreted with caution as we used the overall composite outcome to increase the statistical power in assessing the role of ACEI/ARB in these subgroups. Another point to be noted regarding our subgroup analysis is that the proportion of patients taking ARBs was notably higher than that of patients taking ACEIs, as the regional hypertension management guideline in Korea recommends ARBs over ACEIs due to more favorable adherence and less frequent adverse events [23].

Our study provides clinical evidence indicating that ACEI/ARB use was not associated with a poor prognosis of COVID-19. We generated practicable evidence that addresses an urgent public health need given the uncertainty of clinical consequences of ACEI/ARB use among patients with COVID-19. Second, our results have solid external validity, since they were generated from a completely enumerated cohort of COVID-19 cases that occurred in Korea. Korea has implemented rigorous screening, contact tracing, and quarantine measures, conducting a total of 601,660 COVID-19 screening tests as of April 27, 2020 to proactively contain COVID-19 [24]. All individuals who have epidemiologic links with suspected or confirmed COVID-19 patients or who have arrived from abroad have been required to self-quarantine for 14 days, and those who developed a fever (37.5°C and above) or respiratory symptoms received COVID-19 screening tests; thus, underdiagnosis of COVID-19 is likely to be minimal. Third, our study results were consistent in a subgroup analysis according to the presence of pre-existing diabetes or CVD, which suggests the robustness of our results from confounding by indication given that ACEIs and ARBs are primarily prescribed for patients with these coexisting comorbidities.

Our study also has some limitations. First, the potential misclassification of diagnosis-based outcomes (sepsis and respiratory events) may have occurred. Nevertheless, a validation study comparing diagnoses in the Korean healthcare database with electronic medical records reported an overall positive predictive value of 82% [25]. Death records and procedure codes including mechanical ventilation and ICU admission have high validity, and this issue is thus unlikely to affect our conclusions. Second, there is a potential risk of exposure misclassification owing to a short exposure ascertainment period. However, we found consistent results with the main analysis when the exposure risk window was redefined as 180 days. Third, residual confounding from unmeasured confounders (e.g., smoking history, body mass index, baseline blood pressure, and laboratory test results) may have affected our results given the inherent limitation of available variables in the analysis of health claims data. Finally, we included prevalent users of ACEI/ARB, whereas ideally a new-user design is recommended, in which all study subjects are naïve to previous use of ACEIs/ARBs to address the potential under-ascertainment of events that occur early in therapy and to precisely control for confounders that may be altered by the study drug [26]. However, we used a prevalent user cohort for ACEIs/ARBs given that a new-user design would exclude a large number of subjects representing a clinically relevant subset.

In conclusion, our study findings did not identify an increased risk of adverse outcomes associated with the use of ACEIs or ARBs among COVID-19 patients with hypertension. We present clinical evidence to support current medical societies’ recommendations on continuing ACEIs or ARBs as prescribed in COVID-19 patients.

## Figures and Tables

**Table 1.** Characteristics of ACEI/ARB users and non-users among coronavirus disease 2019 (COVID-19) patients with hypertension

Characteristics	Unweighted, n (%)			IPTW^1^, %		
	ACEI/ARB (n=682)^2^	Non-use (n=608)	aSD	ACEI/ARB^2^	Non-use	aSD
Age (yr)	62.8±14.4	61.3±16.6	0.09	63.4±23.9	62.3±22.6	0.05
<40	42 (6.2)	72 (11.8)		3.9	8.7	
40-64	325 (46.7)	257 (42.3)		50.8	46.0	
65-84	273 (40.0)	244 (40.1)		38.8	39.6	
≥85	42 (6.2)	35 (5.8)		6.6	5.7	
Sex			0.07			0.02
Male	364 (53.4)	303 (49.8)		54.9	53.9	
Female	318 (46.6)	305 (50.2)		45.1	46.1	
Medical history						
Hyperlipidemia	263 (38.6)	204 (33.6)	0.10	41.2	35.5	0.12
Diabetes	252 (37.0)	156 (25.7)	0.25	43.3	33.5	0.20
Cancer	62 (9.1)	76 (12.5)	0.11	7.3	10.7	0.12
Respiratory disease	222 (32.6)	210 (34.5)	0.04	31.8	32.9	0.02
Asthma	59 (8.7)	73 (12.0)	0.11	7.1	10.1	0.11
COPD	137 (20.1)	146 (24.0)	0.10	17.8	21.4	0.09
Bronchiectasis	10 (1.5)	5 (0.8)	0.06	2.2	1.0	0.10
Pneumonia	83 (12.2)	60 (9.9)	0.07	13.7	11.8	0.06
Interstitial lung disease	8 (1.2)	8 (1.3)	0.01	1.0	1.2	0.02
Cardiovascular disease	190 (27.9)	158 (26.0)	0.04	27.3	26.5	0.02
Peripheral vascular disease	64 (9.4)	49 (8.1)	0.05	9.5	9.9	0.01
Coronary artery disease	72 (10.6)	78 (12.8)	0.04	8.8	12.9	0.13
Atrial fibrillation	19 (2.8)	21 (3.5)	0.04	2.8	3.1	0.02
Valvular heart disease	8 (1.2)	2 (0.3)	0.10	2.5	0.7	0.15
Heart failure	59 (8.7)	41 (6.7)	0.07	10.0	7.5	0.09
Arrhythmia	18 (2.6)	20 (3.3)	0.04	2.2	2.8	0.04
Chronic kidney disease	128 (18.8)	95 (15.6)	0.08	18.5	21.0	0.06
Chronic liver disease	80 (11.7)	84 (13.8)	0.06	10.7	13.2	0.08
Stroke	63 (9.2)	74 (12.2)	0.10	7.6	10.5	0.10
Other cerebrovascular diseases	44 (6.5)	43 (7.1)	0.03	5.9	6.6	0.03
Comedications						
CCBs	228 (33.4)	248 (40.8)	0.15	26.5	38.9	0.27
Diuretics	149 (21.9)	133 (21.9)	0.00	22.0	23.2	0.03
β-blockers	182 (26.7)	186 (30.6)	0.09	23.8	29.7	0.14
α-blockers	106 (15.5)	81 (13.3)	0.06	16.5	15.9	0.02
CHA_2_DS_2_-VASc score	2.7±1.4	2.4±1.7	0.35	2.8±2.3	2.5±2.3	0.33
0-1	148 (21.7)	223 (36.7)		14.9	27.4	
2-5	507 (74.3)	353 (58.1)		81.9	67.9	
6-9	27 (4.0)	32 (5.3)		3.3	4.7	
Duration of hypertension (yr)			0.33			0.31
<1	40 (5.9)	74 (12.2)		3.8	8.5	
≥1 and <3	106 (15.5)	141 (23.2)		11.0	19.2	
≥3 and <5	149 (21.9)	141 (23.2)		21.1	20.6	
≥5	387 (56.7)	252 (41.5)		64.2	51.7	

Values are presented as mean±standard deviation or number (%). ACEIs, angiotensin-converting enzyme inhibitors; ARBs, angiotensin II receptor blockers; IPTW, inverse probability of treatment weighting; aSD, absolute standardized difference; COPD, chronic obstructive pulmonary disease; CCBs, calcium channel blockers.

1 To generate IPTW, age at cohort entry, sex, income level, CHA2DS2-VASc score, medical history (including diabetes, cardiovascular disease, stroke, other cerebrovascular disease, hyperlipidemia, respiratory disease, chronic kidney disease, cancer, thromboembolism, and dementia), comedications (including other antihypertensives (CCBs, diuretics, β-blockers, and α-blockers), antidiabetics, antibiotics, antiarrhythmics, antiplatelets, anticoagulants, lipid-lowering agents, and antianginal agents), dialysis, and duration of hypertension (<1, ≥1 and <3, ≥3 and <5, and ≥5 years) were used (c-statistics: 0.723 for ACEI/ARB users vs. non-users).

2 There were 37 (5.4%) ACEI users, 642 (94.1%) ARB users, and 3 (0.4%) ACEI and ARB users.

**Table 2.** RRs of clinical outcomes in ACEI/ARB users compared to non-users among coronavirus disease 2019 (COVID-19) patients with hypertension

Variables	No. of patients	Events, n (%)	Unweighted model		IPTW model^1^	
			Crude RR (95% CI)	Adjusted RR (95% CI)^2^	Crude RR (95% CI)	Adjusted RR (95% CI)^2^
All-cause mortality, mechanical ventilation, ICU admission, sepsis						
Non-use	608	28 (4.6)	1.00 (reference)	1.00 (reference)	1.00 (reference)	1.00 (reference)
ACEIs or ARBs use	682	23 (3.4)	0.73 (0.42, 1.27)	0.71 (0.41, 1.24)	0.60 (0.42, 0.85)	0.60 (0.42, 0.85)*
All-cause mortality						
Non-use	608	12 (2.0)	1.00 (reference)	1.00 (reference)	1.00 (reference)	1.00 (reference)
ACEIs or ARBs use	682	10 (1.5)	0.74 (0.32, 1.72)	0.71 (0.31, 1.67)	0.64 (0.37, 1.12)	0.62 (0.35, 1.09)
Respiratory events^3^						
Non-use	608	108 (17.8)	1.00 (reference)	1.00 (reference)	1.00 (reference)	1.00 (reference)
ACEIs or ARBs use	682	126 (18.5)	1.04 (0.80, 1.35)	1.02 (0.79, 1.32)	0.99 (0.84, 1.16)	0.99 (0.84, 1.17)

RR, relative risk; ACEIs, angiotensin-converting enzyme inhibitors; ARBs, angiotensin II receptor blockers; IPTW, inverse probability of treatment weighting; CI, confidence interval; ICU, intensive care unit.

1 To generate IPTW, age at cohort entry, sex, income level, CHA₂DS₂-VASc score, medical history (including diabetes, cardiovascular disease, stroke, other cerebrovascular disease, hyperlipidemia, respiratory disease, chronic kidney disease, cancer, thromboembolism, and dementia), comedications (including other antihypertensives [calcium channel blockers, diuretics, β-blockers, and α-blockers], antidiabetics, antibiotics, antiarrhythmics, antiplatelets, anticoagulants, lipid-lowering agents, and antianginal agents), dialysis, and duration of hypertension (<1, ≥1 and <3, ≥3 and <5, and ≥5 years) were used.

2 For the adjusted RR, multivariable Poisson regression was used and adjusted for age, sex, CHA2DS2-VASc score, diabetes, cardiovascular disease, and respiratory disease.

3 Respiratory events included acute respiratory distress syndrome, interstitial lung disease, pneumonia, and respiratory failure. *p<0.05.

**Table 3.** Sensitivity analysis with a redefined exposure risk window of 180 days preceding cohort entry for the RRs of clinical outcomes in ACEI/ARB users compared to non-users among coronavirus disease 2019 (COVID-19) patients with hypertension

Variables	No. of patients	Events, n (%)	Unweighted model		IPTW model^1^	
			Crude RR (95% CI)	Adjusted RR (95% CI)^2^	Crude RR (95% CI)	Adjusted RR (95% CI)^2^
All-cause mortality, mechanical ventilation, ICU admission, sepsis						
Non-use	523	23 (4.4)	1.00 (reference)	1.00 (reference)	1.00 (reference)	1.00 (reference)
ACEIs or ARBs use	767	28 (3.7)	0.83 (0.48, 1.44)	0.79 (0.45, 1.38)	0.68 (0.49, 0.94)^*^	0.65 (0.46, 0.90)^*^
All-cause mortality						
Non-use	523	12 (2.3)	1.00 (reference)	1.00 (reference)	1.00 (reference)	1.00 (reference)
ACEIs or ARBs use	767	10 (1.3)	0.57 (0.25, 1.32)	0.52 (0.22, 1.22)	0.45 (0.27, 0.74)^*^	0.41 (0.25, 0.68)^*^
Respiratory events^3^						
Non-use	523	92 (17.6)	1.00 (reference)	1.00 (reference)	1.00 (reference)	1.00 (reference)
ACEIs or ARBs use	767	142 (18.5)	1.05 (0.81, 1.37)	1.08 (0.77, 1.32)	0.98 (0.84, 1.13)	0.94 (0.81, 1.09)

RR, relative risk; ACEIs, angiotensin-converting enzyme inhibitors; ARBs, angiotensin II receptor blockers; IPTW, inverse probability of treatment weighting; CI, confidence interval; ICU, intensive care unit.

1 To generate IPTW, age at cohort entry, sex, income level, CHA2DS2-VASc score, medical history (including diabetes, cardiovascular disease, stroke, other cerebrovascular disease, hyperlipidemia, respiratory disease, chronic kidney disease, cancer, thromboembolism, and dementia), comedications (including other antihypertensives [calcium channel blockers, diuretics, β-blockers, and α-blockers], antidiabetics, antibiotics, antiarrhythmics, antiplatelets, anticoagulants, lipid-lowering agents, and antianginal agents), dialysis, and duration of hypertension (<1, ≥1 and <3, ≥3 and <5, and ≥5 years) were used.

2 For the adjusted RR, multivariable Poisson regression was used and adjusted for age, sex, CHA2DS2-VASc score, diabetes, cardiovascular disease, and respiratory disease.

3 Respiratory events included acute respiratory distress syndrome, interstitial lung disease, pneumonia, and respiratory failure.

* p<0.05.
